# A Large Galenic Dural Arterio‐Venous Fistula in an Adult Managed Surgically: A Case Report

**DOI:** 10.1155/cris/6662325

**Published:** 2026-04-22

**Authors:** Soumya Pahari, Paawan Bahadur Bhandari, Purushottam Baniya, Vinayak Dhungana, Nirvik Gurung

**Affiliations:** ^1^ Neurosurgical Unit, Department of Surgery, Shree Birendra Hospital, Kathmandu, Nepal; ^2^ Department of Surgery, Nepalese Army Institute of Health and Sciences, Kathmandu, Nepal, naihs.edu.np

**Keywords:** galenic dural arteriovenous fistulas, healthcare access, microsurgical interruption of fistula, obstructive hydrocephalus, vascular malformations

## Abstract

Galenic dural arteriovenous fistulas (GDAVFs) are rare, high‐risk vascular malformations within the vein of Galen (VoG), commonly acquired due to factors like venous thrombosis, trauma, or infection and often resulting in severe complications such as hemorrhage or neurological deficits. A 55‐year‐old female presented with pulsatile tinnitus and headaches, with computed tomography (CT) angiography revealing a Borden type III GDAVF with obstructive hydrocephalus. She underwent microsurgical interruption of the fistula via a posterior interhemispheric approach, achieving significant nidus reduction, though a residual fistula persisted. At 3‐month follow‐up, the patient remained asymptomatic. This case underscores the challenges of GDAVFs, where their deep anatomical location complicates treatment, often necessitating a combination of endovascular and microsurgical approaches. Moreover, it highlights the impact of socioeconomic factors on healthcare access, particularly in resource‐limited settings, and emphasizes the need for tailored, multidisciplinary strategies to optimize outcomes.

## 1. Introduction

Galenic dural arteriovenous fistulas (GDAVFs) are a rare and distinct type of arteriovenous fistula that occurs at the wall of the vein of Galen (VoG), a deep cerebral vein critical for venous drainage from the brain. These fistulas are part of the falcotentorial dural arteriovenous fistulas group and are known for their aggressive nature, with almost 97% of cases resulting in severe complications such as hemorrhage or neurological deficits. GDAVFs are considered acquired vascular malformations, commonly developing secondary to factors like venous thrombosis, head trauma, or infections, which increase venous pressure and irreversibly open physiological arteriovenous shunts within the vascular wall of the VoG [1].

The clinical presentation of GDAVFs can be varied, with symptoms including pulsatile tinnitus, headaches, and other signs of intracranial venous hypertension. Advanced imaging modalities such as MRI and computed tomography (CT) cerebral angiography are essential for diagnosing these lesions, revealing characteristic features like dilated veins, aneurysmal changes, and the presence of arterial feeders [2]. The deep anatomical location of the VoG and the intricate nature of the vascular connections make the treatment of GDAVFs particularly challenging. Endovascular techniques, although commonly attempted, often do not achieve complete obliteration of the fistula, necessitating additional microsurgical or radio‐surgical interventions [3]. This paper reports a rare case of an adult GDAVF managed with microsurgical intervention in a resource‐limited setting where financial constraints limit access to endovascular therapy.

## 2. Case Presentation

**Figure 1:**
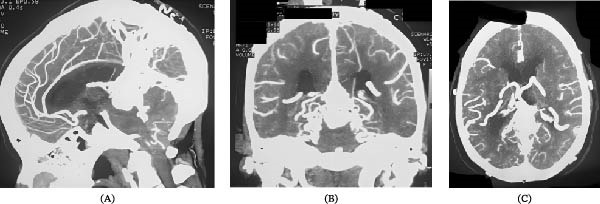
Contrast‐enhanced CT demonstrating a dilated midline venous pouch in the region of the vein of Galen draining into the straight sinus. (A) Sagittal: midline vascular structure draining into the straight sinus. (B) Coronal: dilated venous channel with multiple arterial feeders. (C) Axial: prominent tortuous vessels consistent with high‐flow arteriovenous shunting.

**Figure 2:**
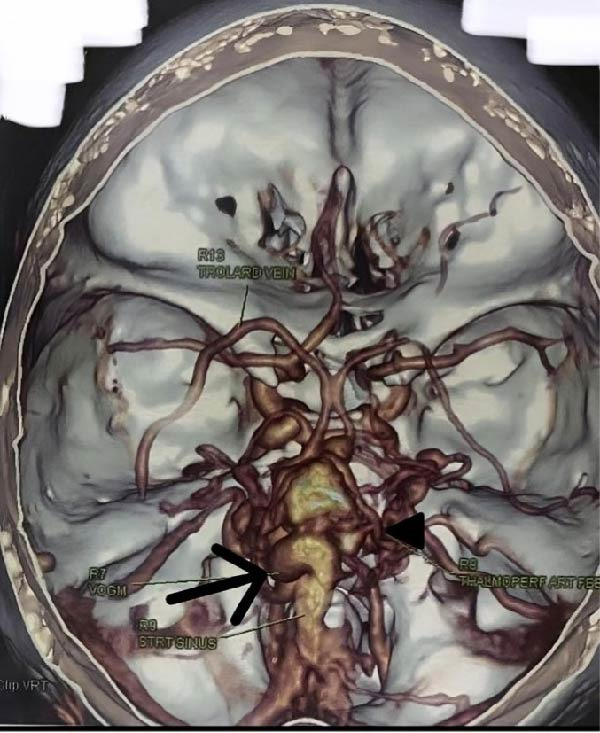
3‐Dimensional computed tomography (CT)—cerebral angiogram: aneurysmal dilatation and tortuosity of VoG with arterial feeders from the thalamoperforating and posterior‐choroidal arteries.

A 55‐year‐old female presented with pulsatile tinnitus and persistent headaches of 1 month’s duration. She denied any history of fever, vomiting, trauma, visual disturbances, or ear complaints. On examination, no significant findings were noted. Contrast‐enhanced CT indicated a dilated VoG, and subsequent CT cerebral angiography showed aneurysmal dilatation with tortuous VoG and arterial feeders from the thalamoperforating and posterior choroidal arteries. Bilateral internal cerebral veins were also dilated, and the nidus was observed to compress the cerebral aqueduct (Figures 1 and 2). The diagnosis was consistent with a Borden type III Galenic DAVF, accompanied by obstructive hydrocephalus. The patient was counseled regarding the potential risk of intraoperative fistula rupture, injury to the cerebellum, and cranial nerve palsies.

**Figure 3:**
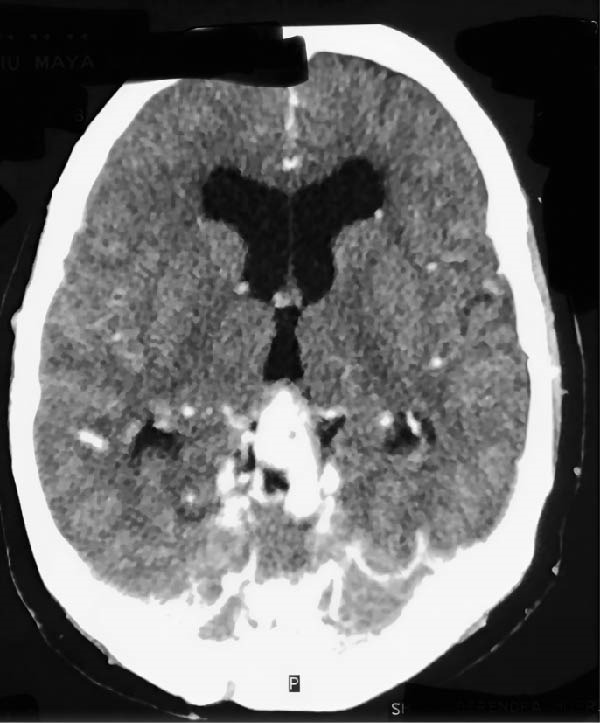
Contrast‐enhanced CT—axial. Postoperative contrast‐enhanced CT performed 2 days after surgery demonstrating reduction in nidus size.

Endovascular intervention was not available at our institute, and the patient was unable to afford a referral to a neurointervention center. The patient underwent microsurgical interruption of the fistula via a posterior interhemispheric approach. Intraoperatively, the dura was extremely vascular. Intraoperative indocyanine green angiogram was used to assess the vascular anatomy of the malformation. The major arterial feeders identified were the falcine artery and the bilateral occipital arteries. Microsurgical interruption of the fistula was done. Following this, repeat ICG angiography demonstrated marked reduction of early venous filling within the dilated VoG, suggesting effective disconnection of the dominant arterial feeder. Both intraoperative and postoperative courses were uneventful. Follow‐up imaging demonstrated a significant reduction in the nidus size, though a residual fistula persisted (Figure 3). Due to financial constraints, the patient declined any further vascular imaging or repeat surgical intervention. At a 3‐month follow‐up, she remained asymptomatic despite declining further intervention due to financial constraints.

## 3. Discussion

GDAVFs in adults are complex vascular lesions that differ significantly from congenital VoG aneurysmal malformations seen in pediatric patients. The pathophysiology of GDAVFs involves the abnormal connection between arterial and venous systems at the VoG, leading to increased venous pressure and subsequent vascular complications [4]. These fistulas are generally acquired due to conditions that elevate venous pressure, such as venous thrombosis, trauma, or infections. However, in some cases, like the one presented, no clear precipitating factors are identified, which adds to the complexity and challenges of managing these cases [5].

The primary goals in treating GDAVFs are to prevent catastrophic hemorrhage and to alleviate symptoms caused by intracranial venous hypertension. Symptoms like pulsatile tinnitus, headaches, and cognitive decline are often related to the elevated venous pressure and reduced cerebral perfusion associated with these fistulas [6]. The deep‐seated location of the VoG and the complex anatomy of the arteriovenous connections present significant challenges for surgical intervention [7]. Endovascular treatment, which involves the use of coils, liquid embolic agents, or stents, is frequently employed but often proves insufficient due to incomplete obliteration of the fistula and the high risk of periprocedural bleeding [2]. In recent studies of complex Dural venous fistulas, it has been observed that it often does not achieve complete obliteration in complex fistulas due to multiple high‐flow arterial feeders [8].

Microsurgical intervention, as demonstrated in this case, offers a more definitive treatment option, particularly when endovascular approaches are inadequate [3]. The posterior interhemispheric approach used in this case allowed for direct access to the fistula, facilitating effective interruption of the abnormal arteriovenous connections. Despite the successful reduction in the size of the nidus, the persistence of the fistula highlights the need for ongoing monitoring and potentially further treatment [4]. Financial constraints and patient preferences also play a critical role in the management decisions, as seen with the patient’s refusal of additional surgery, one of the major barriers to healthcare access in Nepal [9].

This case underscores the importance of a multidisciplinary approach in diagnosing and managing GDAVFs, integrating advanced imaging techniques, endovascular expertise, and microsurgical skills to optimize patient outcomes. The rarity and complexity of GDAVFs necessitate continued research and collaboration to develop more effective treatment strategies and improve the prognosis for affected patients [1]

## 4. Conclusion

GDAVFs in adults are rare, complex vascular malformations associated with a high hemorrhagic risk, posing significant therapeutic challenges. Microsurgical intervention represents a feasible and effective treatment in cases when endovascular therapy is not available. This case illustrates the critical importance of a tailored, multidisciplinary approach, especially in resource‐limited environments where socioeconomic factors may limit treatment options.

## Funding

This research did not receive any specific grant from funding agencies in the public, commercial, or not‐for‐profit sectors.

## Disclosure

The authors are accountable for all aspects of the work in ensuring that questions related to the accuracy or integrity of any part of the work are appropriately investigated and resolved.

## Ethics Statement

All procedures performed in this study were per the ethical standards of the institutional and national research committee(s) and with the Helsinki Declaration (as revised in 2013). Written informed consent was obtained from the patient for the publication of this case report and accompanying images. All the patients allowed personal data processing, and informed consent was obtained from all individual participants included in the study. A copy of the written consent is available for review by the editorial office of this journal. The case report is done based on the SCARE Checklist.

## Conflicts of Interest

The authors declare no conflicts of interest.

## Data Availability

The data that support the findings of this study are available upon request from the corresponding author. The data are not publicly available due to privacy or ethical restrictions.
